# Genome-wide DNA methylation profiling is able to identify prefibrotic PMF cases at risk for progression to myelofibrosis

**DOI:** 10.1186/s13148-021-01010-y

**Published:** 2021-02-04

**Authors:** Ulrich Lehmann, Helge Stark, Stephan Bartels, Jerome Schlue, Guntram Büsche, Hans Kreipe

**Affiliations:** Institute of Pathology, Medical School Hannover, Medizinische Hochschule Hannover, Carl-Neuberg-Str. 1, 30625 Hannover, Germany

**Keywords:** DNA methylation, 850 k EPIC array, Myelofibrosis, Prefibrotic PMF

## Abstract

**Background:**

Patients suffering from the *BCR-ABL1*-negative myeloproliferative disease prefibrotic primary myelofibrosis (pre-PMF) have a certain risk for progression to myelofibrosis. Accurate risk estimation for this fibrotic progression is of prognostic importance and clinically relevant. Commonly applied risk scores are based on clinical, cytogenetic, and genetic data but do not include epigenetic modifications. Therefore, we evaluated the assessment of genome-wide DNA methylation patterns for their ability to predict fibrotic progression in PMF patients.

**Results:**

For this purpose, the DNA methylation profile was analyzed genome-wide in a training set of 22 bone marrow trephines from patients with either fibrotic progression (*n* = 12) or stable disease over several years (*n* = 10) using the 850 k EPIC array from Illumina. The DNA methylation classifier constructed from this data set was validated in an independently measured test set of additional 11 bone marrow trephines (7 with stable disease, 4 with fibrotic progress). Hierarchical clustering of methylation *β-*values and linear discriminant classification yielded very good discrimination between both patient groups. By gene ontology analysis, the most differentially methylated CpG sites are primarily associated with genes involved in cell–cell and cell–matrix interactions.

**Conclusions:**

In conclusion, we could show that genome-wide DNA methylation profiling of bone marrow trephines is feasible under routine diagnostic conditions and, more importantly, is able to predict fibrotic progression in pre-fibrotic primary myelofibrosis with high accuracy.

## Introduction

Primary myelofibrosis (PMF) belongs to the *BCR/ABL1*-negative myeloproliferative neoplasms and is often characterized by a bi-phasic course of disease. In the initial phase, the disease manifests itself with thrombocytosis and granulocytosis without bone marrow fibrosis [1, 2]. This phase can last for years (stable disease). In a considerable subset of cases, progression takes place leading to bone marrow fibrosis, extramedullary hematopoiesis with splenomegaly, and frequently thrombocytopenia. The duration of stable disease and the risk to fibrotic progression varies substantially between individual patients [3]. Progressive bone marrow fibrosis is a life-threatening condition and, hematopoietic stem cell transplantation (HSCT) provides the only curative therapy. Considering the considerable risk connected with HSCT [4, 5], the proper identification of patients with higher risk of fibrotic progression is of utmost importance.

So far the identification of molecular risk factors with overall survival or blastic transformation as endpoints has focused on karyotype, gene expression, and genetic alterations [6–8]. Recently, we have analyzed the association of fibrotic progression in PMF cases with age-related clonal hematopoiesis (ARCH) [9]. Another age-related phenomenon in hematopoietic cells is the alteration of epigenetic patterns, mainly gene methylation [10]. Surprisingly little is known about the role of epigenetic aberrations in this context. DNA methylation, the best known and best understood epigenetic mechanism which is altered in many pathological conditions [11, 12], is analyzed only in a few studies about MPNs. Some early studies employing in part now outdated methodology analyzed the DNA methylation of only single genes [13–15]. More comprehensive approaches employing Illumina’s 27 k array or 450 k array methodology focused on the differences between MPN patients and healthy controls or the differences between MPN subtypes [16–18]. None of these studies addressed the relationship between epigenetic alterations and progression from a pre-fibrotic stage to overt myelofibrosis.

Therefore, we analyzed the DNA methylation profile in bone marrow trephines from patients with pre-fibrotic PMF at diagnosis with no indications for excess of blasts or marrow fibrosis (EB0 and MF0) who later developed myelofibrosis (MF2 or 3). The results were compared with the DNA methylation patterns in bone marrow trephines from prefibrotic PMF patients who showed stable disease for at least four years after diagnosis. For this purpose, the most recent version of the Illumina DNA methylation array was employed (850 k EPIC array).

## Results

### Suitability of routinely processed FFPE bone marrow trephines for genome-wide DNA methylation analyses

In a first step, we analyzed 4 samples using the Illumina EPIC array in order to figure out whether the amount and quality of genomic DNA extracted from the routinely processed, fixed, decalcified, and embedded bone marrow trephines in our institution provide a reliable and evaluable signal output.

Altogether, 658,746 out of 865,859 CpG sites were evaluable (see “Material and Methods” section for details). That means, only 14% of the CpG under showed a signal to noise ratio too low for proper evaluation, demonstrating the feasibility of EPIC DNA methylation array analyses of FFPE bone marrow trephines. This offers the unique opportunity to combine molecular studies with histomorphological examination. Other groups could demonstrate this for different tissue types as well [19–21].

The amount of genomic DNA used for DNA methylation analysis and the age of the sample (both are important variables under routine conditions and for archival specimens) could be excluded as confounding factors in our series of altogether 33 samples (using the Singular Value Decomposition algorithm in ChAMP and a *p*-value cutoff of 0.05 [22], see Additional file 1: Figure S1).

### Genome-wide DNA methylation profiling identifies patients suffering from fibrotic progression

In order to establish a DNA methylation classifier for fibrotic progression, a training set of 22 samples (10 displaying stable disease, 12 fibrotic progression) was used (for inclusion criteria see Materials and Methods section). A test set of additional 11 samples (7 displaying stable disease, 4 fibrotic progression) was used in a second step for independent confirmation of the methylation classifier.

**Fig. 1:**
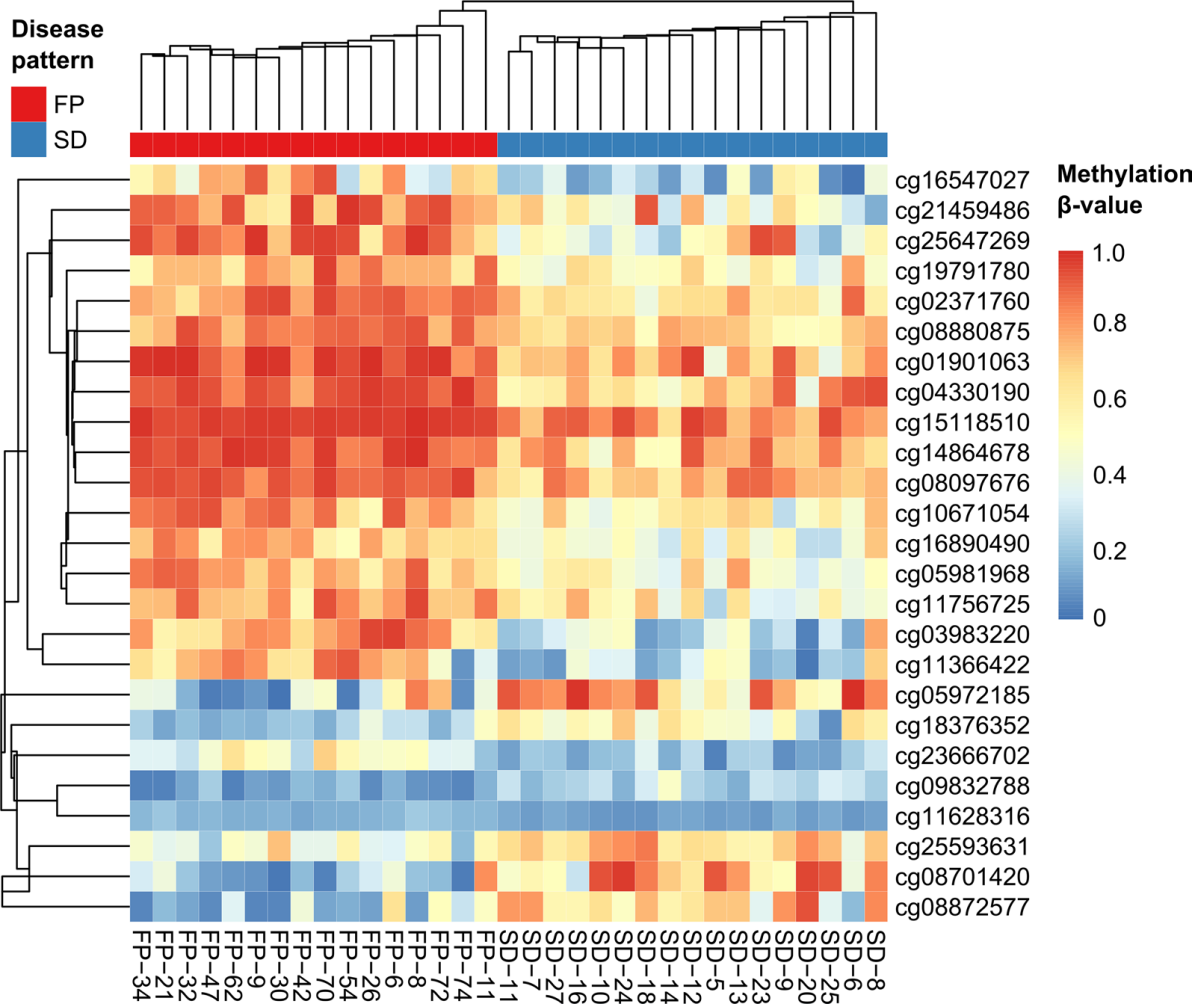
Hierarchical clustering heatmap of differentially methylated probes. Centroid clustering was used as linkage criterion. Shown are all significantly differentially methylated probes between samples with fibrotic progression (FP) and those with stable disease (SD) at an FDR threshold of 0.1. Additional file 12: Table S3 contains the exact location of all 25 CpG sites. Additional file 3: Figure S3 shows the hierarchical clustering for training and test cohort separately.

Unsupervised hierarchical clustering of the 1000 most differentially methylated CpG sites yielded a very good separation of both patient groups. Figure 1 shows the heatmap for the 25 most differentially methylated CpG sites for the combined training and test set. Patients displaying fibrotic progression are clearly separated from the patients with stable disease. Since all samples were taken from patients in the hypercellular early pre-fibrotic phase dominated by granulopoiesis and megakaryopoiesis the cellular composition, which is later changing over the course of the disease and may influence DNA methylation patterns, can be excluded as confounding factor. Additional file 2: Figure S2 displays the clustering for the training and the test set separately and Additional file 3: Figure S3 displays the results for the 1000 most differentially methylated regions.

### Identification of a minimal set of differentially methylated CpG sites

**Fig. 2:**
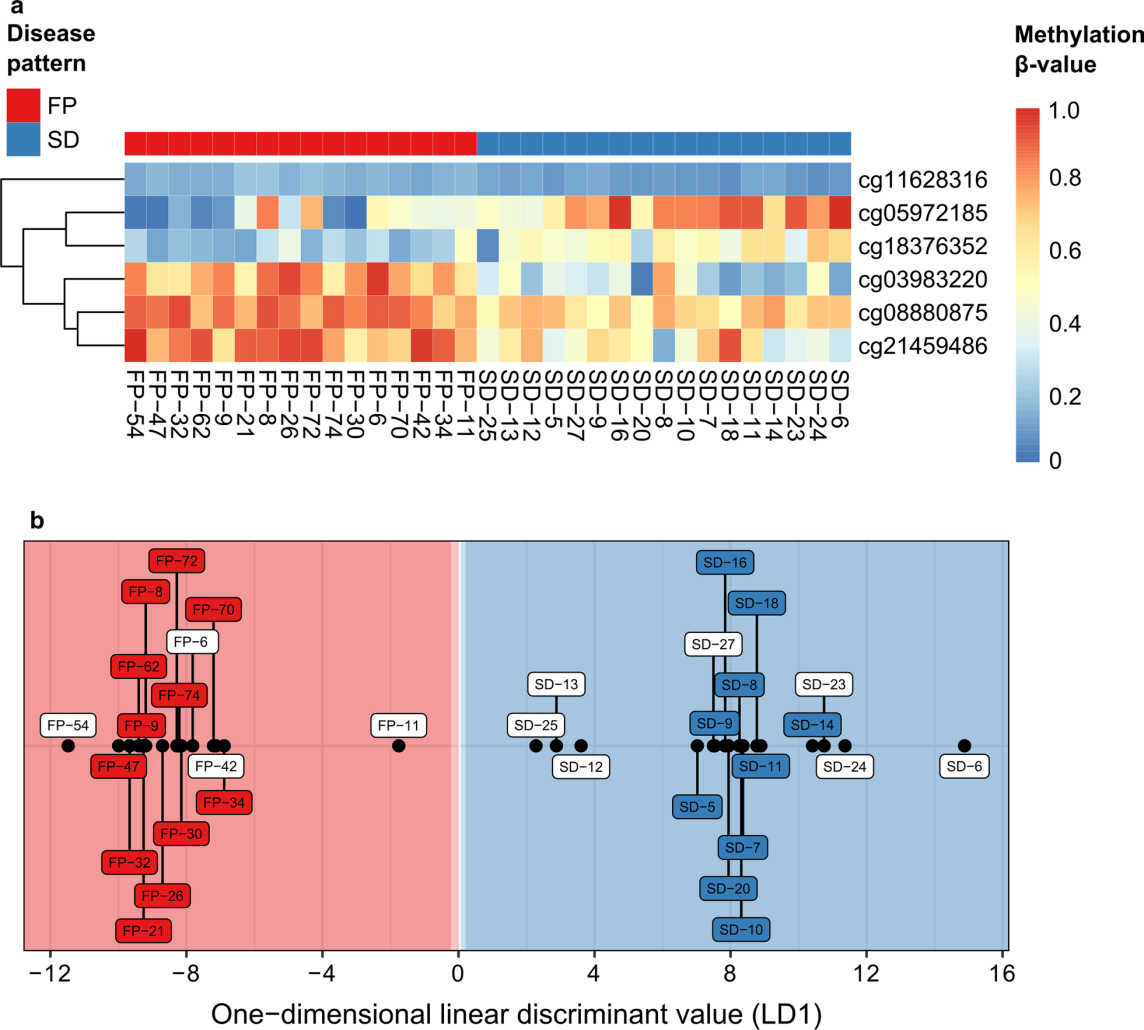
**a** Heatmap of methylation *β-*values for the 6 differentially methylated CpG sites used for classification analysis. The 6 CpG sites shown are required and sufficient for perfect linear discriminant classification of samples with fibrotic progression (FP) and stable disease (SD). The order of samples equals their class membership probability and is identical to the order of samples in **b**. Additional file 4: Figure S4 shows the results of the hierarchical clustering based on these 6 CpG sites separately for the training and test cohort. **b** Linear discriminant classification of samples based on 6 differentially methylated CpG sites. Linear discriminant values (LD1) show the predicted class membership of samples with fibrotic progression (FP, left) and stable disease (SD, right). Colored background areas indicate regions of high confidence (dark, class probability ≥ 95%) or lower confidence (bright, class probability > 50%). The linear discriminant classifier was trained using methylation *β-*values from 6 CpG sites of 22 samples (colored sample labels). Subsequent model validation was performed against 11 independently measured samples (white sample labels).

Further analysis of the differentially methylated CpG sites revealed that smaller subsets could be used to discriminate samples displaying fibrotic progress from patients with stable disease, the minimum being 6 CpG sites (Fig. 2a, see also Additional file 4: Figure S4 for 100% separation of the SD and FP cohort in the training and test set separately based on only these 6 CpG sites). In the next step, linear discriminant analysis (LDA) [23] was performed using the above described training set off 22 samples and the minimal subset of 6 CpG sites. The resulting classifier was subsequently validated against 11 independently measured samples (7 with stable disease, 4 with fibrotic progress) and yielded very good accuracy against both training and test set. Figure 2b shows the perfect discrimination between the two groups.

Table 1 lists the exact localization and the functional annotation of the 6 CpG sites which are required and sufficient for discrimination of the two patient groups as well as their coefficients for the linear discriminant model. Four of the six CpG sites are associated with known genes, coding for transcription factors as well as proteins involved in cell division and cytokine signaling. A gene set enrichment analysis against the Gene ontology (GO) database based on the 25 most differentially methylated probes yielded no statistically relevant results. Therefore, we used differentially methylated regions as input instead, which revealed a striking enrichment of the biological functions “cell–cell and cell–matrix adhesion” (see Additional file 5: Table S1 and Additional file 6: Table S2).

**Table 1:** Genomic localization and functional annotation of the 6 CpG sites shown in Fig. 2a

Chr	Name	Localization	Associated gene		Coefficient for LDM
16	cg03983220	OpenSea	None		− 7.431161
10	cg11628316	Island	NM_001008541	Max-interacting protein 1	− 44.910325
2	cg08880875	N_Shore	NM_138804	Meiosis 1 Associated Protein	− 11.469252
13	cg21459486	OpenSea	none		− 9.140643
16	cg05972185	OpenSea	NM_001164766	Zinc finger homeobox 3	6.017707
19	cg18376352	Island	NM_004750	Cytokine Receptor-Like Factor 1	12.946803

For the definition and nomenclature of CpG islands, shores, open sea, and shelves, see [49, 50]

*LDM* Linear discriminant model

### Analysis of suboptimal samples

Initially, 7 samples (out of 40) failed the stringent quality metrics evaluation process. Three out of these 7 samples missed the thresholds of various parameters only minimally, thereby representing suboptimal samples which can be encountered under routine diagnostic conditions.

Additional file 7: Figure S5 demonstrates that these 3 samples are correctly classified if the LDA classification algorithm established with the training and test set is applied. These promising results should be confirmed with more suboptimal samples for further demonstration of the usefulness of our newly developed DNA methylation classifier for routine diagnostic analyses.

### Hypo- and hypermethylation in stable disease versus fibrotic progression

The heat maps in Figs. 1 and 2a) already indicate by visual inspection that fibrotic progression is associated with hypermethylation (*β-*values with red color code) relative to the samples from patients with stable disease. A more detailed analysis of the differentially methylated CpG sites confirms this visual impression: The mean *β-*value (± standard error) of all 25 methylated CpG sites in the fibrotic progression group is 0.64 ± 0.02, versus 0.52 ± 0.01 in the stable disease group (*p* = 1.3e−12, Mann–Whitney-*U* test). Additional file 8: Figure S6 illustrates in a more global view the gain in hypermethylated loci (*β-*value > 0.8) in the cohort showing fibrotic progression compared to the group with stable disease. Additional file 9: Figure S7 and Additional file 10: Figure S8 shows this in more detail (Additional file 9: Figure S7) and in a more quantitative way (Additional file 10: Figure S8). However, hyper- and hypomethylation take place in both patient cohorts simultaneously in a complex way.

### Copy number alterations and fibrotic progress

In a next step, we investigated whether copy number gains or losses are able to identify patients undergoing fibrotic progress and whether a CNA analysis might support the DNA methylation classifier. Calculating gene copy number alterations from the genome wide DNA methylation data obtained with the EPIC array is a straightforward well established procedure [24].

**Fig. 3:**
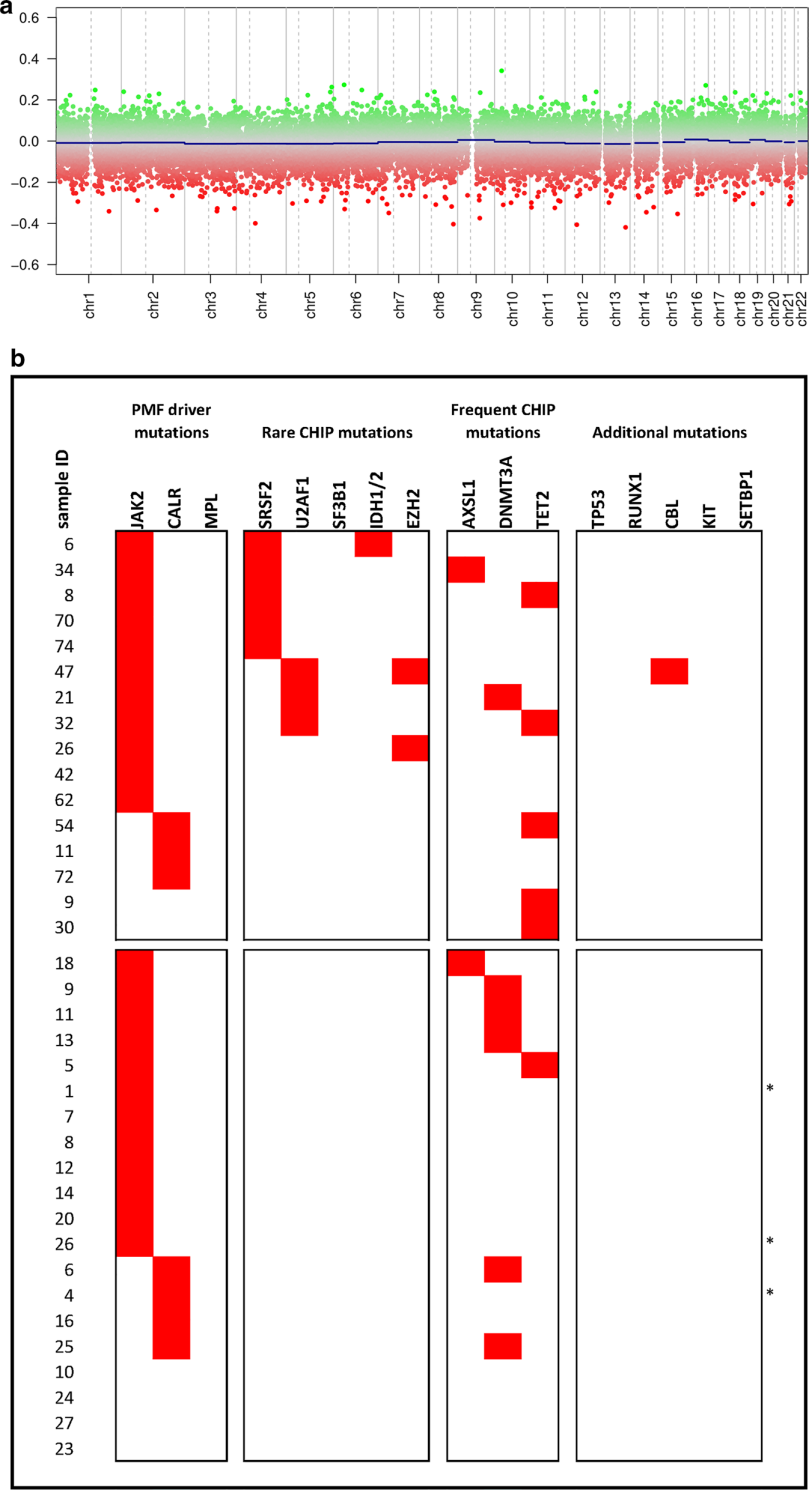
**a** Copy number alterations (CNA) in cases with stable disease versus cases showing progression to myelofibrosis. Log2 copy ratios for individual CpG sites as well as segments were calculated from the median signal intensity of samples with fibrotic progression compared to samples with stable disease as reference set. The *x*-axis visualizes genomic locations with centromere positions indicated by dashed lines, whereas the y-axis indicates individual log2 copy ratios. No gains or losses of segments (absolute log2 copy ratio ≥ 0.5) could be detected between both sample sets. **b** Results of the mutation analysis. The cases showing progress to myelofibrosis are represented in the upper part of the figure, the cases with stable disease in the lower part. Each red square indicates the presence of a mutation causing a loss of function or an altered protein function. The asterisk on the right side (*) mark the three samples which failed the initial QC procedure, but were classified correctly using the linear discriminant classifier (see Additional file 1: Figure S1). Since the case series of this study is a subset of the series described in detail before by us the results of the mutation profiling are also contained within Fig. 2 of Bartels et al.

In Fig. 3a, the median copy number values for each chromosome in the group showing progression to myelofibrosis were calculated using the group with stable disease as a reference. No gains or losses of segments (absolute log2 copy ratio ≥ 0.5) capable of distinguishing the two sample sets could be detected.

### Mutation profiles and fibrotic progress

In recent years, several groups [7, 8, 25] including our own [9] identified mutational signatures associated with fibrotic progress. Despite strong statistically significant correlations comparing groups of patients the predictive power of mutation profiling for individual samples can be improved, especially in the absence of these alterations.

Mutations in *ASXL1*, *EZH2*, *IDH1/2*, and, *SRSF2,* called “high molecular risk, HMR” [26] or “molecular high risk, MHR” [27] mutations, have been associated with worse prognosis in PMF patients. There is a clear statistical significant association of the presence of one or more mutation of this type with fibrotic progress also in our cohort: 7 out of 16 (44%) with fibrotic progress versus 1 out of 20 (5%) with stable disease, Chi^2^-test, *p* = 0.0065. However, 7 out of 16 samples (44%) from patients displaying progress to myelofibrosis do not have any mutation in one of these five genes (Fig. 3b), indicating the limitation of taking only gene mutations into account.

In contrast to the methylation profile, the presence of well-described high molecular risk mutations in *ASXL1*, *EZH2*, *IDH1/2*, or *SRSF2* is not able to predict progression to myelofibrosis (*p* = 0.99 in the multivariate analysis, see below).

### Multivariate analysis of clinical data and methylation scores

For each patient, all available clinical parameters (patient age at diagnosis, patient sex, mutation status, leukocyte count, platelet count, and hemoglobin concentration) as well as the LDA value calculated from the methylation *β-*values of 6 CpG sites were used for an explorative multivariate analysis. The numbers of leukocytes and platelets were only available for 18 out of 33 patients and the concentration of hemoglobin only for 17 out of 33 patients. Therefore, these parameters were modeled independently from the other parameters using Bayesian logistic regression.

All demographic and clinical data including age, sex, leukocyte count, platelet count, and hemoglobin concentration did not show any statistically significant difference between the two cohorts. Only rare CHIP mutations (Fig. 3b) were significant for regression (*p* = 0.0346). However, the LD value calculated for each sample showed a better level of significance in this analysis (*p* = 0.0098) and also allows for accurate classification of samples with fibrotic progression but without rare CHIP mutations (which are frequent but not ubiquitous in samples with fibrotic progression).

### Epigenetic aging and fibrotic progress

It has already been speculated for a long time that DNA methylation aberrations accumulate over the life span of an organism due to the intrinsic error rates of the enzymatic machinery responsible for proper maintenance of these patterns (see [28] and references therein) and that these accumulating DNA methylation aberrations contribute to the development of various diseases including cancer [11, 12]. Developing this idea further, several groups established algorithms based on the quantitative assessment of DNA methylation patterns to measure the epigenetic age (EA) of a given patient sample in comparison with its chronological age (CA) [29]. Discrepancies between the epigenetic and the chronological age might indicate increased risk for developing certain pathological conditions.

Three algorithms have been developed and are widely discussed in the literature: the Horvarth [30], Hannum [31], and PhenoAge [32] signature. In this study, we are not interested in measuring the chronological age of the samples (which is known and documented) by analyzing DNA methylation patterns. Instead, discrepancies between the (known) chronological age and the calculated “epigenetic” age are of interest in our context. These differences might indicate premature aging of patients associated with morbidity. Therefore, PhenoAge is in our context the most suitable approach [29].

**Fig. 4:**
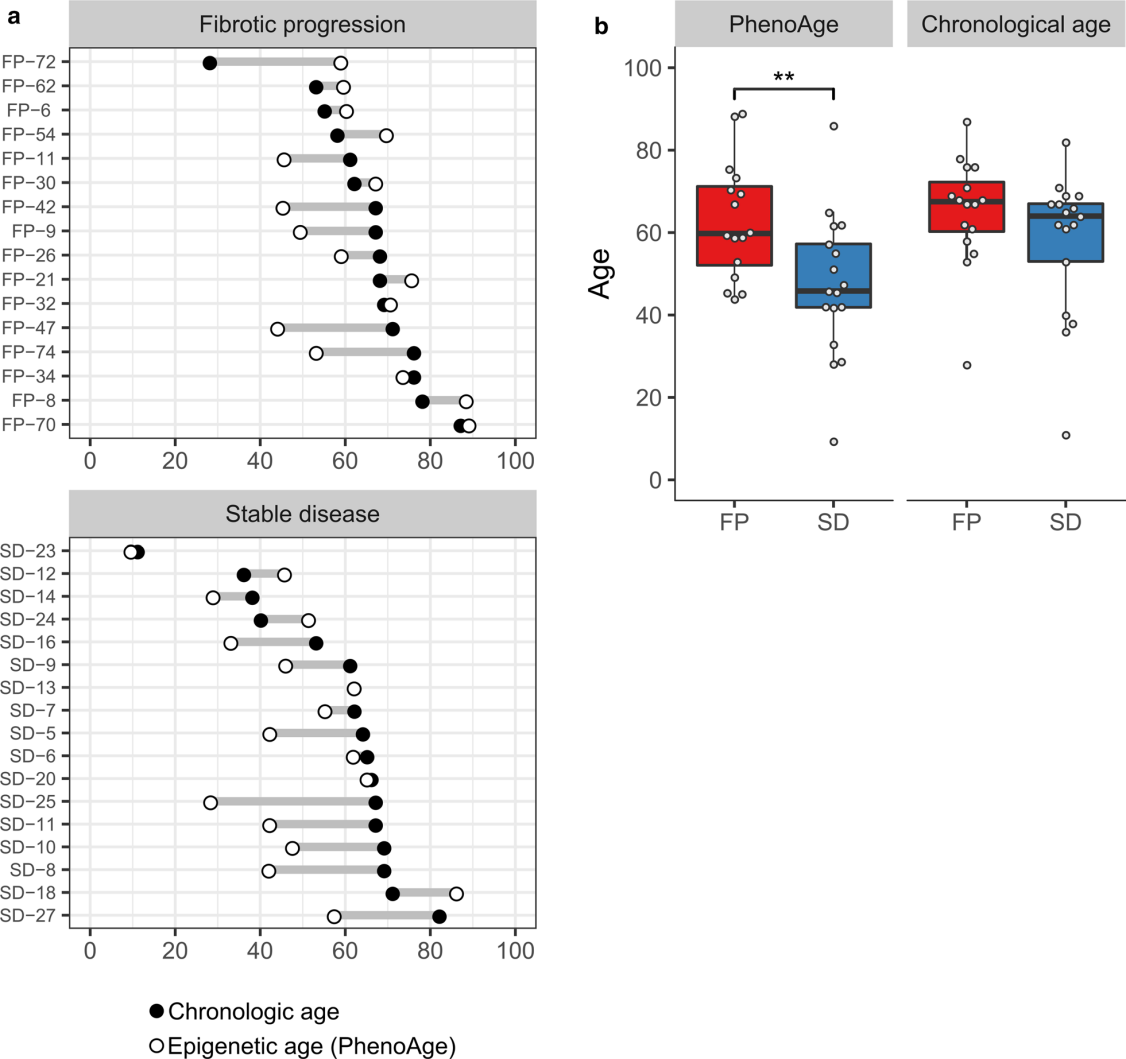
**a** Differences in epigenetic and chronological age per patient. Each line represents an individual patient. The calculated epigenetic age is larger than the chronological age in 9 out of 16 cases with fibrotic progress (65,3%), in the group with stable disease this is the case in only 3 out of 17 cases (17,6%, Chi^2^-test, *p* = 0.021). **b** Differences in epigenetic and chronological age per disease group. Comparing the mean epigenetic age and the mean chronological age between both sample sets it turns out that the mean of the „PhenoAge “ is statistically significantly higher in the group showing fibrotic progression (FP) compared to the stable disease (SD) group (*p* = 0.003 Mann–Whitney-*U* test), whereas the mean chronological age is very similar in both disease groups

Figure 4a shows that in 9 out of 16 cases with fibrotic progress (65.3%), the calculated epigenetic age is higher than the chronological age, whereas for the group with stable disease, this is the case in only 3 out of 17 cases (17.6%, chi^2^-test, *p* = 0.021) indicating an association between risk of progress to myelofibrosis and an increase in epigenetic age. Figure 4b confirms this relationship by calculating differences between mean chronological and mean epigenetic age of both patient groups (not by evaluating and counting individual cases as in Fig. 4a. The calculated epigenetic age in the cohort without progression to fibrosis (SD) is lower than the chronological age in this group, whereas in the cohort with fibrotic progression both are very similar, meaning that the mean calculated epigenetic age is on average higher in the patients who later develop myelofibrosis compared to the patients with stable disease (*p* = 0.003 Mann–Whitney-*U* test).

## Discussion

Analyzing histopathologically well characterized bone marrow trephines from prefibrotic PMF patients, we could show for the first time that the analysis of DNA methylation patterns is able to identify those patients who will progress to myelofibrosis. For this purpose, we employed the EPIC array technology which interrogates the methylation status of 850,000 individual CpG sites.

In comparison with other hematological malignancies, surprisingly little is known about DNA methylation patterns in MPNs, their dynamics during course of disease, and their diagnostic and prognostic impact. In two recent comprehensive reviews about myeloproliferative diseases, aberrations in DNA methylation are not mentioned at all [25, 33].

The review by Kim and Abdel-Wahab entitled “Focus on the epigenome in the myeloproliferative neoplasms” [34] concentrates nearly exclusively on mutations in genes encoding proteins involved in DNA and histone modifications and mentions alterations in these modifications itself (i.e., alterations in DNA methylation) only in passing.

Nischal et al. [35] demonstrated in their pioneering study using HELP assay (*Hpa*II tiny fragment enriched by LM-PCR) that comprehensive DNA methylation profiling is able to distinguish the three *BCR-ABL1*-negative MPN subgroups polycythemia vera (*n* = 8), essential thrombocytosis (*n* = 6), and primary myelofibrosis (*n* = 11). Samples from pre-fibrotic PMF patients later showing progression to myelofibrosis were not analyzed at that time.

The findings from Nischal et al. were confirmed by Perez et al. [16] using the 27 k methylation array from Illumina. Due to the limited resolution of the technology they found mostly differences between (1) MPN samples and healthy controls and (2) MPN samples and MPN samples transformed into AML. Nielsen et al. [17] using the much more comprehensive 450 k array from Illumina focused in their interesting DNA methylation profiling study on different cellular compartments of the hematopoietic system analyzing flow-sorted cells and the relationship between DNA methylation on the one hand and histone modifications and gene mutations on the other hand. The course of disease within individual patients (i.e., progression to myelofibrosis) was not under study.

The interesting association between *ASXL1* mutations and distinct methylation profiles reported by Nielsen et al. [17] could not be confirmed in our study because both our groups included only a single *ASXL1* mutated case each. Also, *DNMT3A* and *TET2* alterations are too rare in our cohort for robust meaningful conclusions. Only the previously reported association of mutations rarely affected by age-related clonal hematopoiesis (ARCH) with progression to fibrosis [9] could be confirmed (see Fig. 4b). However, 6 from 16 patients (37.5%) undergoing progression to myelofibrosis did not show any mutation commonly found in MDS and only rarely in ARCH. Four patients (25%) did not show any mutation in the set of 25 genes tested, indicating certain limitations of this approach in analyzing individual cases. The multivariate analysis of all parameters under study clearly showed that the presence of so-called high risk mutations in *ASXL1*, *EZH2*, *IDH1/2*, or *SRSF2* is not able to predict progression to myelofibrosis (*p* = 0.99).

Also mutations in the tumor suppressor gene *TP53*, incorporated by Grinfeld et al. in their recently published “MPN Personal Risk Score”[36], do not contribute to a separation of the two patient groups in our study, because they are too rare in our prefibrotic PMF cases.

From the fibrotic progression subgroup 7 patients had a high molecular risk mutation (Fig. 3), and 9 patients had an epigenetic age higher than the chronological age (Fig. 4). However, only 3 patients overlap in both groups, supporting the hypothesis that DNA methylation profiles are independent from the mutation profile and provide additional prognostic information.

In a more recent genome-wide DNA methylation profiling project (also using Illumina’s 450 K array), Martinez-Calle et al. [18] identified only differences between patients with myelofibrosis and healthy controls, similar to the findings reported earlier by Perez et al. [16]. No differences within the group of patients with myelofibrosis were reported, and the question of fibrotic progress has also not been addressed in this study.

Comparing DNA methylation profiles from healthy controls and patients with a certain disease or from patients with different diseases always comes with the risk of confounding factors distorting the DNA methylation profiles, the most important being cellular composition of the blood or the bone marrow, respectively (see [37] and references therein). Therefore, we compared only DNA methylation profiles from patients with the same disease and disease stage, i.e., prefibrotic PMF (MF0 and EB0). The only difference between the two groups is the later course of disease (i.e., with and without progression to myelofibrosis). This focus on patients diagnosed with prefibrotic PMF (EB0, MF0) avoids all confounding factors distorting the DNA methylation profiles.

The influence of the epigenetic age on the course of disease in the context of myeloproliferative disease has also been studied by McPerson et al.[38]. However, they analyzed only patients with polycythaemia vera (PV) and essential thrombocythaemia (ET), before and after treatment with the histone deacetylase inhibitor vorinostat, finding opposite effects in both diseases. They used the three-gene aging signature proposed by Weidner et al.[39] for analyzing changes in DNA methylation during treatment. Therefore, the interesting results of this study and the results presented here cannot be compared directly.

The gene ontology analysis identified biological processes and molecular functions related to cell–cell and cell–matrix adhesion as highly enriched in the group of differentially methylated genes (see Additional file 5: Table S1), indicating that these processes might be more important for disease progression than, e.g., proliferation or cell death (apoptosis). In other organs, like liver or lung, the role of adhesion molecules for the development of fibrosis is already well studied [40, 41]. Future functional studies have to elucidate the role of the differentially methylated regions identified herein for the progression to myelofibrosis in PMF patients.

A limitation of the present study is the modest sample size which should be increased in follow-up studies. Multi-center collaborations will be necessary to collect much larger numbers of appropriate samples with complete clinical and histopathological records.

## Conclusions

This study compares, to the best of our knowledge, for the first time the DNA methylation profile in pre-fibrotic PMF patients who later develop overt myelofibrosis with the DNA methylation profile from pre-fibrotic PMF patients not progressing to myelofibrosis. This approach identifies the prognostic potential of comprehensive DNA methylation profiling for patients presenting with prefibrotic PMF. In addition, the importance of cell–cell and cell–matrix interactions for these processes is highlighted by the enrichment in the gene ontology analysis and should be followed-up in future studies.

The technique used in this study is already well established in other diagnostic settings (see [24, 42–44] as examples) and should be implementable with reasonable efforts after independent validation in a larger cohort.

## Materials and methods

### Patient samples

Decalcified formalin-fixed and paraffin-embedded bone marrow trephines collected between 2000 and 2018 were retrieved retrospectively from the archive of the Institute of Pathology, Hannover Medical School. All cases underwent histopathological routine diagnostic procedures. For the cohort displaying progression to fibrosis (FP), cases with two consecutive biopsies and a minimum of 1 year follow-up time (range: 1–12.5 years, mean: 4 years, median: 2 years) were selected. The first bone marrow biopsy at the time of initial diagnosis had to be classified as prefibrotic PMF without increase in blasts (EB0) according to WHO definition [45] and with no signs of myelofibrosis (MF0). The second biopsy showed clear progression to myelofibrosis (MF 2 or 3). For the stable disease (SD) cohort without development of fibrosis, a minimum of 4 years follow-up time (range: 4–14 years, mean: 7.3 years, median: 6 years) was required with clinically and histologically persisting prefibrotic PMF. The first and last biopsies in this cohort were MF grade 0 and EB grade 0. For further details, see Table 2. In this cohort without progression to fibrosis, the reasons for performing subsequent bone-marrow biopsies were quite heterogeneous, e.g., anemia or thrombosis. (for details see ref [9]).

**Table 2:** Clinical parameters

	Progression to fibrosis (FP)	Stable disease (SD)
	*n* = 16	*n* = 20
Females	6 (38%)	11 (55%)
Age (at diagnosis, mean)	62 years	56 years
Total follow-up	75.5 years	154 years
Range	1.0–12.5 years	4.0–14.0 years
Mean follow-up	5 years	7.3 years
Hemoglobin (g/dL, median)	11.7	14.8
Leukocytes (× 10^6^ × L^−1^)	16.5	9.2
Thrombocytes (× 10^9^ × L^−1^)	776	912
Spleen size (mean)	13.4 cm	14.3

The cohort analyzed in this study represents a subgroup of the cohort described in detail in Bartels et al. 2020 [9]. The selection criteria for the training set and the validation set were the availability of a sufficient amount of DNA (therefore, not all samples from Bartels et al. could be included in this study) and a balanced ratio between cases with stable disease and progression to fibrosis, respectively.

The study design is following the guidelines of the Hannover Medical School ethics committee for retrospective analyses.

### DNA extraction

Extraction of genomic DNA was performed using the Maxwell RSC instrument (Promega, Madison, WI, USA) and the Maxwell RSC DNA FFPE kit according to the manufacturer's instructions. Three to five sections of 10 µm thickness each were taken, depending on the size of the trephine. After extraction, nucleic acid concentrations were quantified using a Qubit 2.0 fluorimeter (ThermoFisherScientific, Carlsbad, CA, USA) and the Qubit dsDNA high sensitivity kit (ThermoFisherScientific, Carlsbad, CA, USA).

### DNA methylation analysis

For genome-wide DNA methylation analyses, the 850 k EPIC array platform from Illumina was used [46]. At least 250 ng genomic DNA extracted from FFPE bone marrow trephines was used. In the majority of cases, 500 ng genomic DNA were available. Bisulfite conversion, array hybridization, and raw data collection were done at Life&Brain (Bonn, Germany) following the manufacturer’s protocols. Subsequently, raw data files were downloaded from this company’s server for further analyses. The 850 K EPIC array data series are deposited in the Gene Expression Omnibus (GEO) data base (GEO accession number: GSE152519). Array data analysis was performed with R (version 3.4.4). The R package IlluminaHumanMethylationEPICanno.ilm10b3.hg19 (version 0.6.0) was used for chip annotation. The R package MethylAid (version 1.12.0) was used for quality control (default settings) [47]. The R package ChAMP (version 2.9.10) was used for data loading, sample exclusion, and batch effect correction (all default settings) as well statistical analyses and intra-sample probe normalization via the BMIQ algorithm [48, 49]. The batch variable "AMP_Plate" was designated as confounder and the variable "Sample_Class" as predictor for batch correction via the ComBat algorithm [50]. No batch correction was performed on data used for machine learning and modeling. Differentially methylated probes (DMPs, representing individual CpG sites) were identified using the limma algorithm included in the ChAMP package with a false discovery rate (FDR) threshold of ≤ 0.1 [51]. Therefore, in this study the definition of “differentially methylated” is solely driven by statistical analyses (via the FDR) and not by a *β-*value threshold. Gene set enrichment analysis (GSEA) against the Gene Ontology (GO) database was performed based on the differentially methylated regions (DMR) between both groups, which were identified using the Bumphunter algorithm included in the ChAMP package with an FDR of ≤ 0.05 and the parameters ‘maxGap’ and ‘cutoff’ changed from their default values to 250 and 0.99, respectively. The GSEA itself was performed using the gometh algorithm included in the ChAMP package and an FDR threshold of 0.1. The R package “conumee” in bioconductor was employed for identification of copy number variations [52]. For the calculation of the epigenetic methylation age, the PhenoAge algorithm from the R package ENmix (version 1.25.1) was used [32, 53].

### Classification of samples

In order to select suitable DMPs for sample classification, we utilized an exact leaps and bounds algorithm aimed at optimizing the Tau-squared coefficient from the R package sub-select (version 0.14 [54]). The resulting CpG subsets were used to create data sets from the training set of 22 samples as input for the implementation of linear discriminant analysis (LDA) in the R package caret (version 6.0-81 [23]). It is important to point out that in contrast to the data used for statistical analysis; no batch correction was performed on data used for classification of samples. A singular value decomposition analysis shows that ComBat batch correction could be successfully used to reduce the influence of confounding variables (such as AMP_Plate) on the methylation data set (see Additional file 11: Figure S9). For resampling and estimating preliminary model performance, we used fivefold repeated cross-validation. Tuning parameters were left at default settings. All models were finally evaluated concerning their accuracy against a validation set of 11 independently measured samples. When yielding the same accuracy, models were preferred that require less input features (i.e., DMPs).

### Multivariate analysis

In order to deal with the moderate sample sizes and occasional separation by chance, we used Bayesian logistic regression instead of logistic regression for our analysis of demographic and clinical data. Parameters were considered to be significant if their *p*-value for the *Z*-statistic was below 0.05.

### Mutation profiling

Targeted re-sequencing of 23 genes was performed using an amplicon-based NGS panel, and pyrosequencing was used for analysis of *MPL* Codon 515 and for *ETNK1* Codon 244 as described [55].

### Analysis of copy number alterations

The analysis of copy number variations based on their methylation profiles was performed using the R package conumee (version 1.12.0) [52]. For this purpose, the signal intensities of individual samples with fibrotic progression as well as the median signal intensity of these samples was compared to the set of samples with stable disease as a reference (default settings).

## Supplementary Information

**Additional file 1: Figure S1.** Singular value decomposition analysis for identification and exclusion of confounding factors. Sample age and amount of DNA both show a *p*-value above 0.05 in the SVD analysis, demonstrating that these two variables are clearly not confounding factors.

**Additional file 2: Figure S2.** Hierarchical clustering based on 25 CpG sites from Fig. 1 separately for the training and test cohort.

**Additional file 3: Figure S3.** Hierarchical clustering based on 1000 differentially methylated CpG sites and histogram of *β-*values. This figure corresponds to Fig. 1. The lower panel shows the histogram for all *β-*values illustrating the gain in methylation in the fibrotic progression cohort, accompanied also by a loss of DNA methylation in a smaller group of CpG sites.

**Additional file 4: Figure S4.** Hierarchical clustering based on 6 CpG sites from Fig. 2 separately for the training and test cohort.

**Additional file 5: Table S1.** Gene set enrichment analysis of differentially methylated regions against the Gene Ontology database.

**Additional file 6: Table S2.** Detailed description of the 40 most differentially methylated regions (DMR).

**Additional file 7: Figure S5.** Linear discriminant classification of low-quality samples. Three samples (white background labels) which had been excluded from the statistical analysis due to high proportions of probes above the detection *p*-value threshold of 0.1 are classified correctly using the linear discriminant model.

**Additional file 8: Figure S6.** Histogram of all *β-*values with fixed thresholds for hyper- and hypomethylation. With a *β-*value threshold of > 0.8 for hypermethylation and < 0.1 for hypomethylation the simultaneous gain in hyper- and in hypomethylated loci in the fibrotic progression cohort is obvious. The number of CpG sites with very high methylation level (i.e., above 0.8) is larger in the FP cohort compared to the SD cohort (245,829 vs 204,494, or 20.2% more highly methylated sites in the FP cohort) and this increase in highly methylated sites in the FP cohort is three times higher than the increase in CpG sites with very low methylation (i.e., below 0.1) in this cohort (relative to the SD cohort the number of CpG sites with low methylation level increases by only 7.7%). Therefore, it is justified to state that overall the fibrotic progression cohort is characterized by hypermethylation relative to the stable disease cohort.

**Additional file 9: Figure S7.** Boxplots for the *β-*values of the 25 differentially methylated CpG sites shown in Fig. 1. The display of the individual boxplots shows the simultaneous gain and loss of DNA methylation in the fibrotic progression cohort relative to the stable disease cohort (as shown in a more global view in Additional file 8: Figure S6). 18 from 25 CpG sites display a higher methylation level in the FP group compared to the SD group.

**Additional file 10: Figure S8.** Number of differentially methylated CpG sites in the FP group versus the SD group. **a** Number of CpG sites more heavily methylated (“hypermethylated”) in the FP or the SD group at a given threshold for the difference in the *β-*value. **b** Histogram for Δ*β* > 0.1. **c** Histogram for Δ*β* > 0.2. The predominance of more heavily methylated loci in the FP group is obvious. The SD group shows a distribution centered around *β-*values between 0.5 and 0.6, whereas the FP group shows a clear skewing of the distribution towards *β-*values between 0.8 and 0.9.

**Additional file 11: Figure S9.** Singular value decomposition analysis of the batch correction. ComBat batch correction could be successfully used to reduce the influence of confounding variables (such as AMP_Plate) on the methylation data set, leaving “sample class” as the most important discriminator.

**Additional file 12: Table S3.** Detailed description of the 25 most differentially methylated CpG sites at FDR < 0.1.

## Data Availability

The DNA methylation array data are deposited at GEO (GSE152519).
