# Supplementary figures and images for: Genome-wide DNA methylation profiling is able to identify prefibrotic PMF cases at risk for progression to myelofibrosis
Source: Clin Epigenetics. 2021 Feb 4;13:28. doi: 10.1186/s13148-021-01010-y (PMC7860011; doi:10.1186/s13148-021-01010-y)

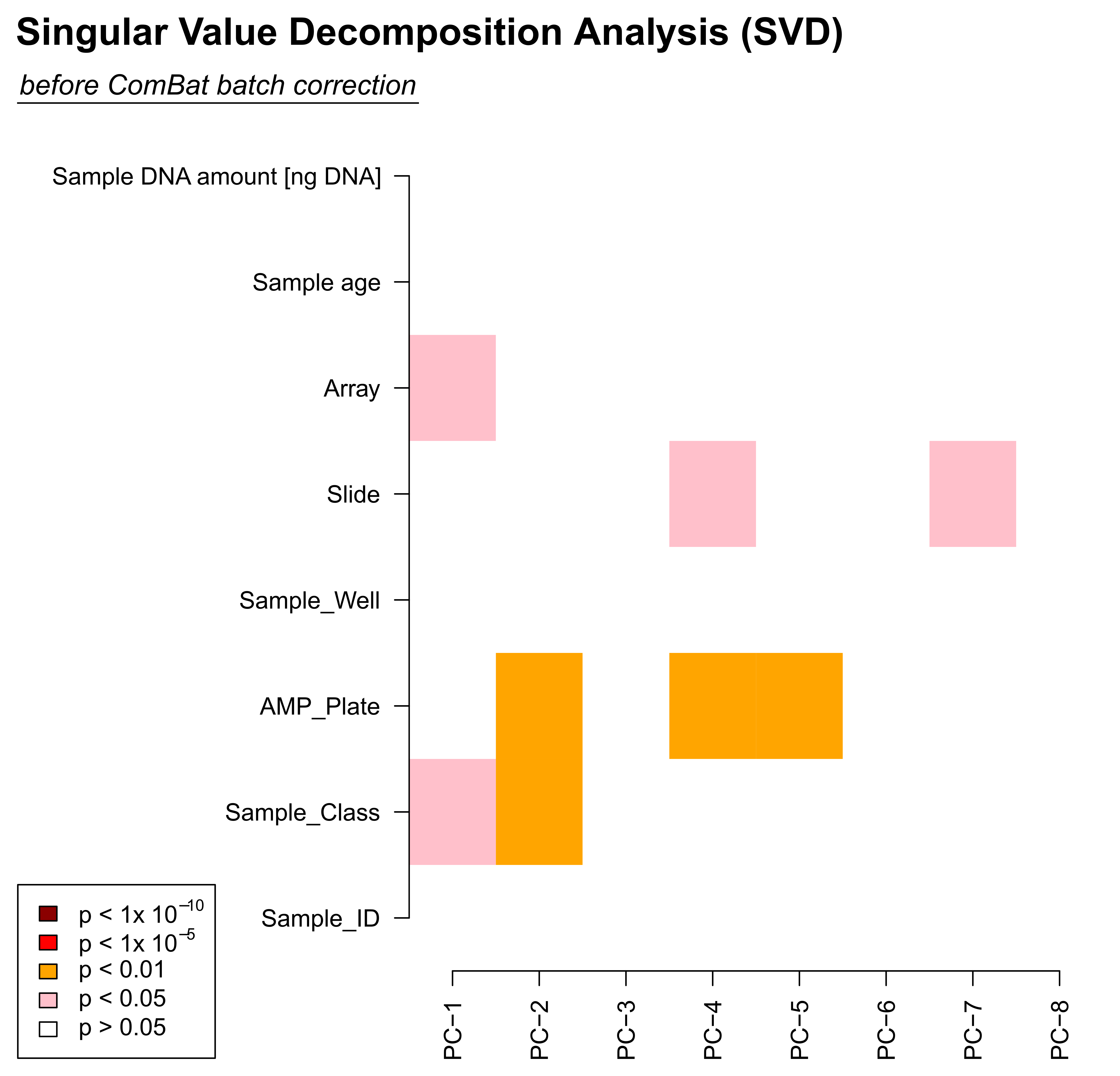

Supplement: Supplementary file 1 — Additional file 1: Figure S1. Singular value decomposition analysis for identification and exclusion of confounding factors. Sample age and amount of DNA both show a p-value above 0.05 in the SVD analysis, demonstrating that these two variables are clearly not confounding factors. [file 13148_2021_1010_MOESM1_ESM.tif]

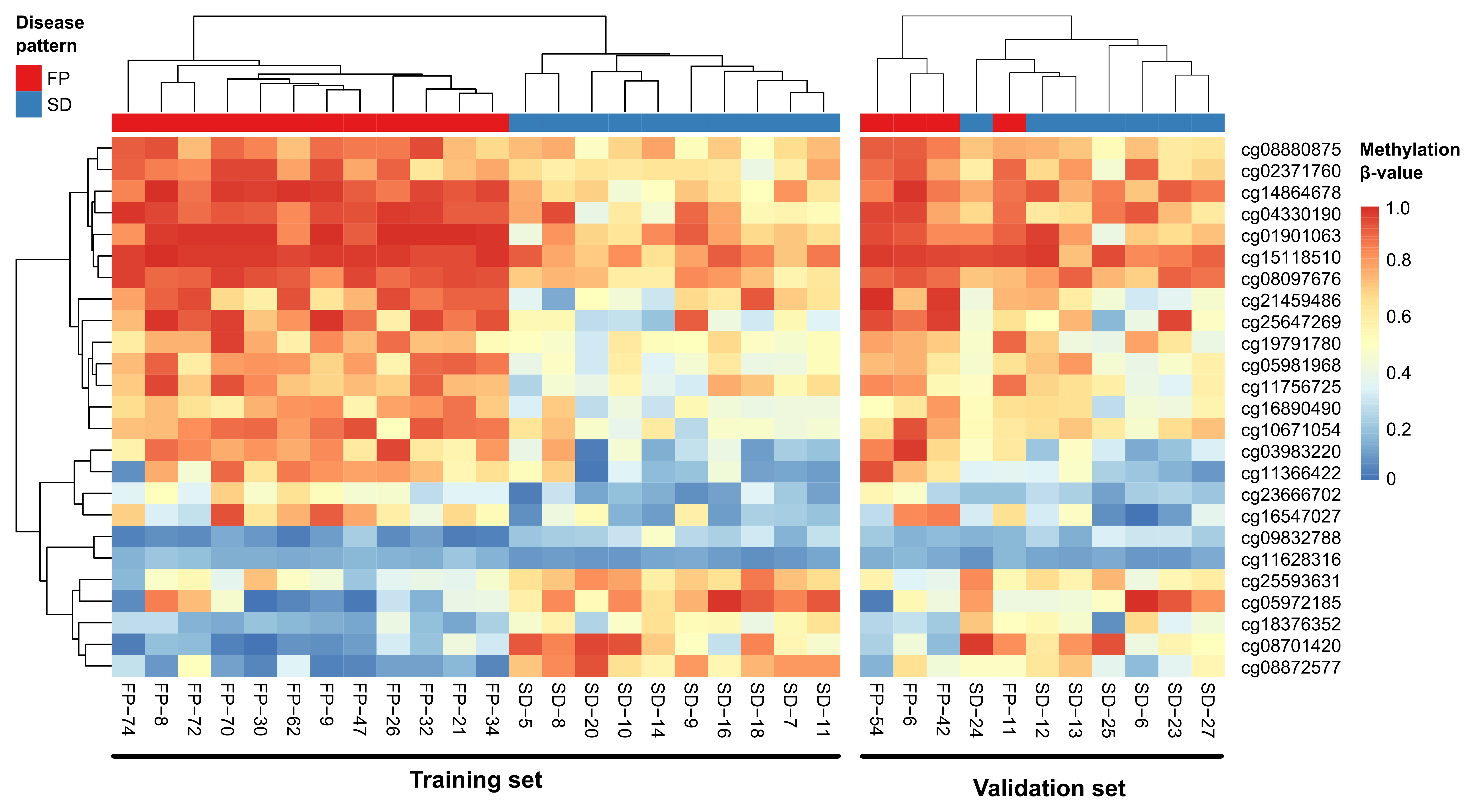

Supplement: Supplementary file 2 — Additional file 2: Figure S2. Hierarchical clustering based on 25 CpG sites from Fig. 1 separately for the training and test cohort. [file 13148_2021_1010_MOESM2_ESM.tif]

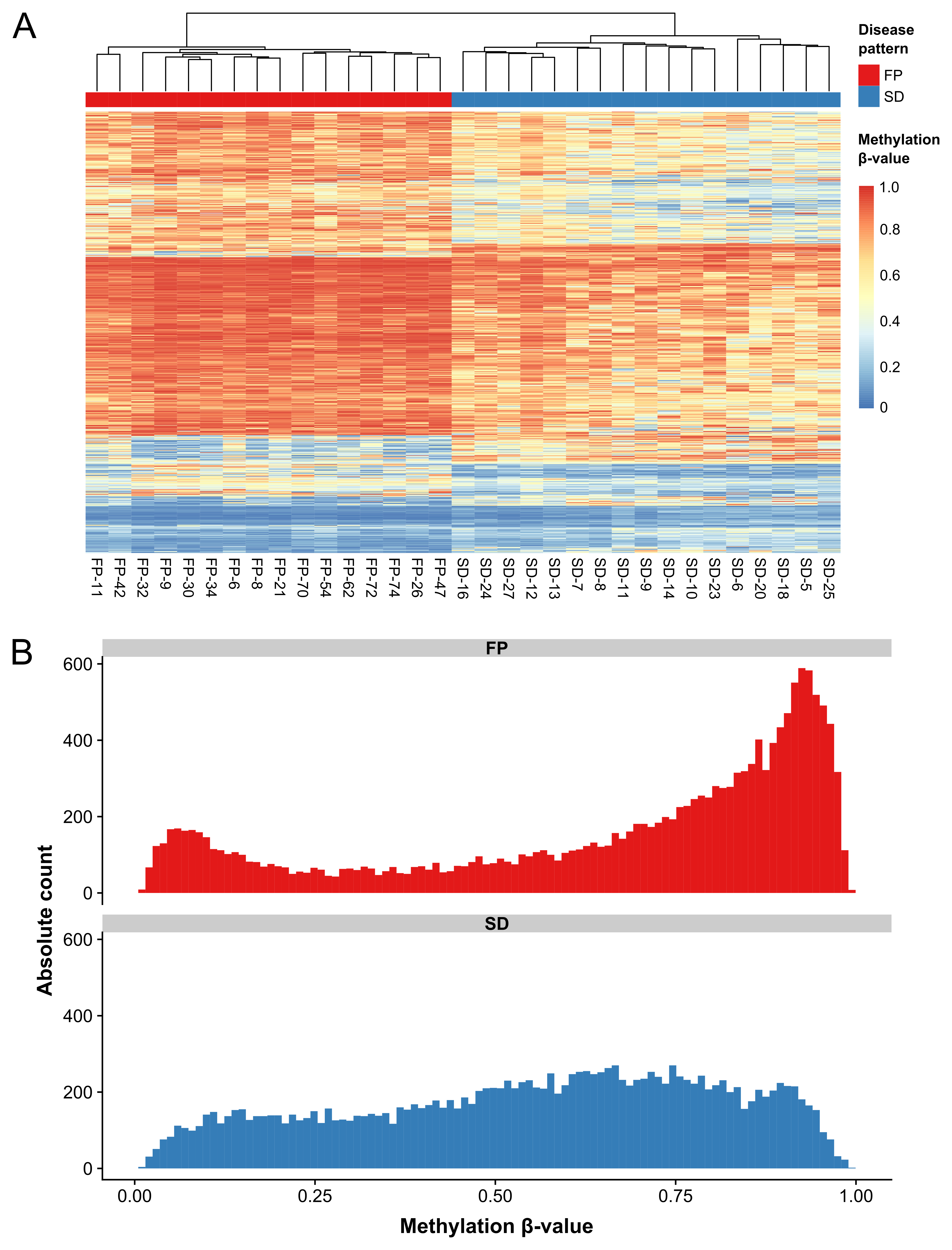

Supplement: Supplementary file 3 — Additional file 3: Figure S3. Hierarchical clustering based on 1000 differentially methylated CpG sites and histogram of β-values. This figure corresponds to Fig. 1. The lower panel shows the histogram for all β-values illustrating the gain in methylation in the fibrotic progression cohort, accompanied also by a loss of DNA methylation in a smaller group of CpG sites. [file 13148_2021_1010_MOESM3_ESM.tif]

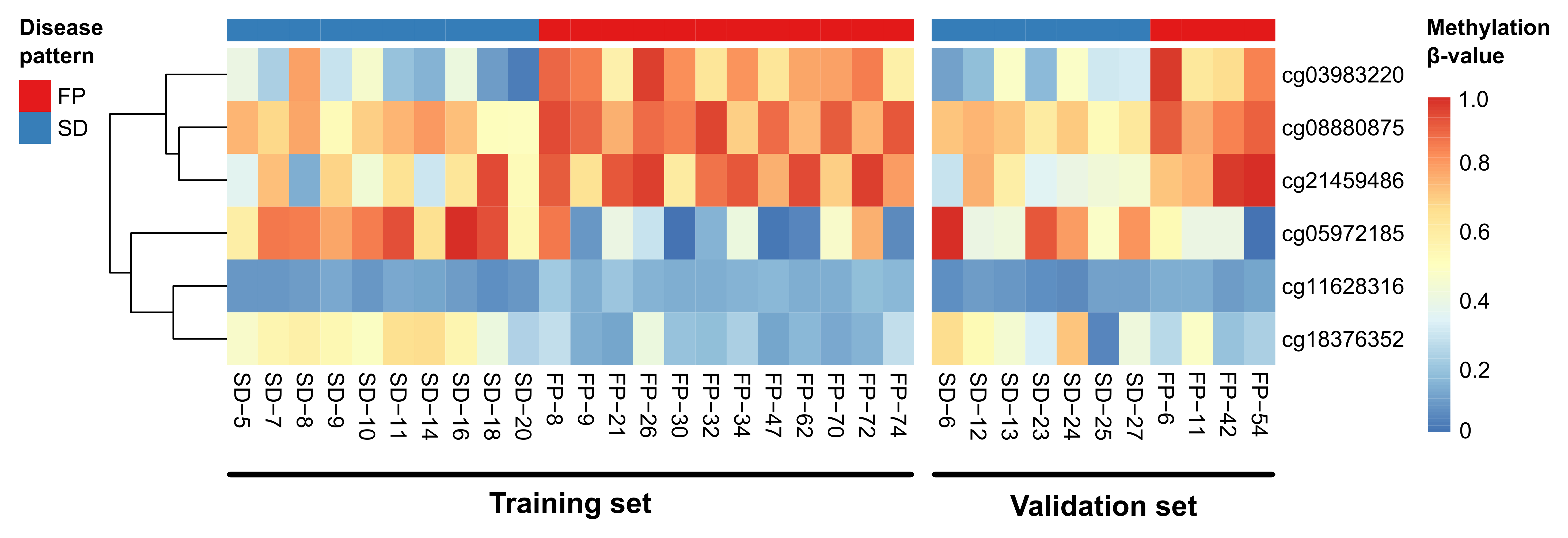

Supplement: Supplementary file 4 — Additional file 4: Figure S4. Hierarchical clustering based on 6 CpG sites from Fig. 2 separately for the training and test cohort. [file 13148_2021_1010_MOESM4_ESM.tif]

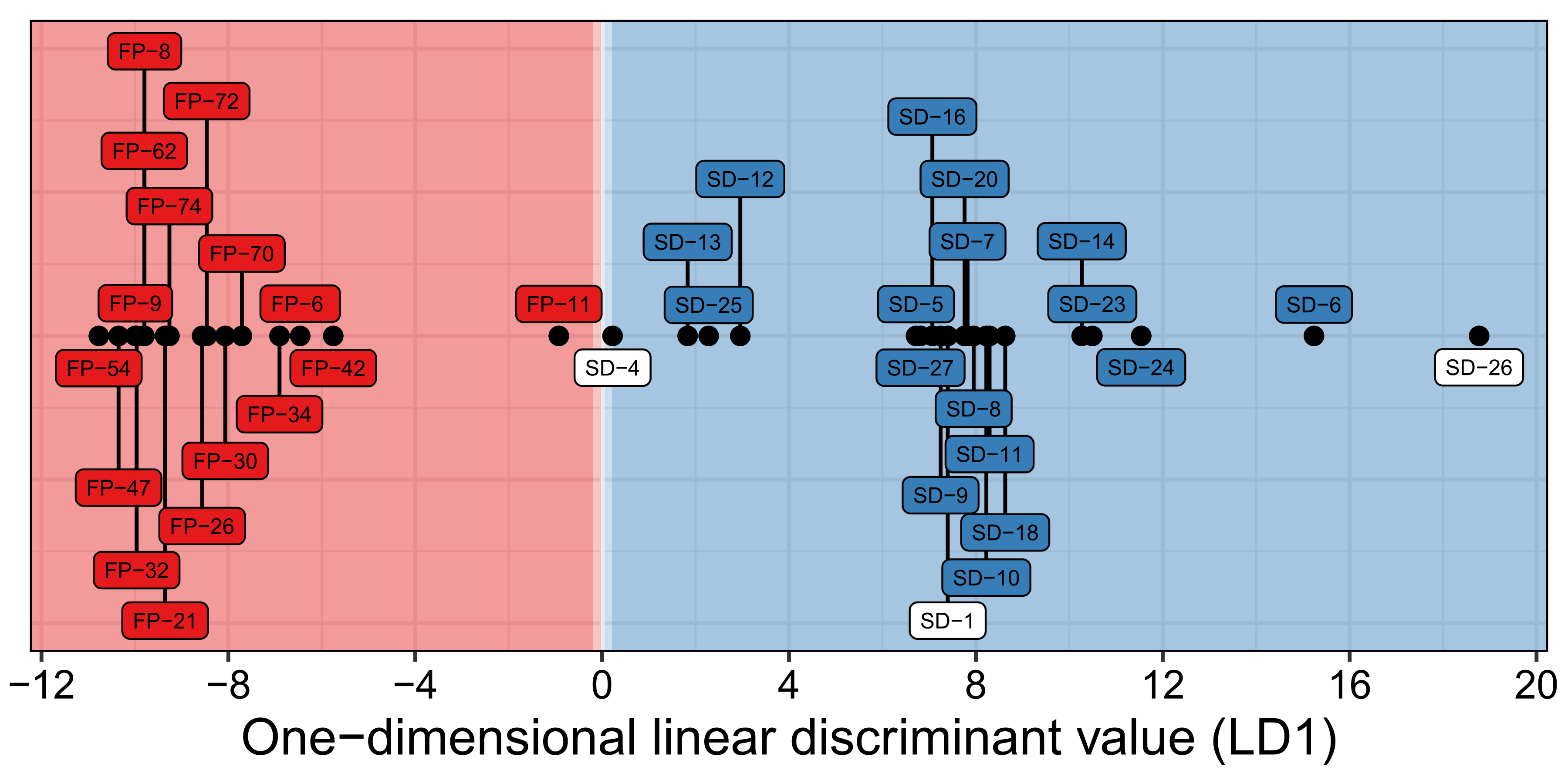

Supplement: Supplementary file 7 — Additional file 7: Figure S5. Linear discriminant classification of low-quality samples. Three samples (white background labels) which had been excluded from the statistical analysis due to high proportions of probes above the detection p-value threshold of 0.1 are classified correctly using the linear discriminant model. [file 13148_2021_1010_MOESM7_ESM.tif]

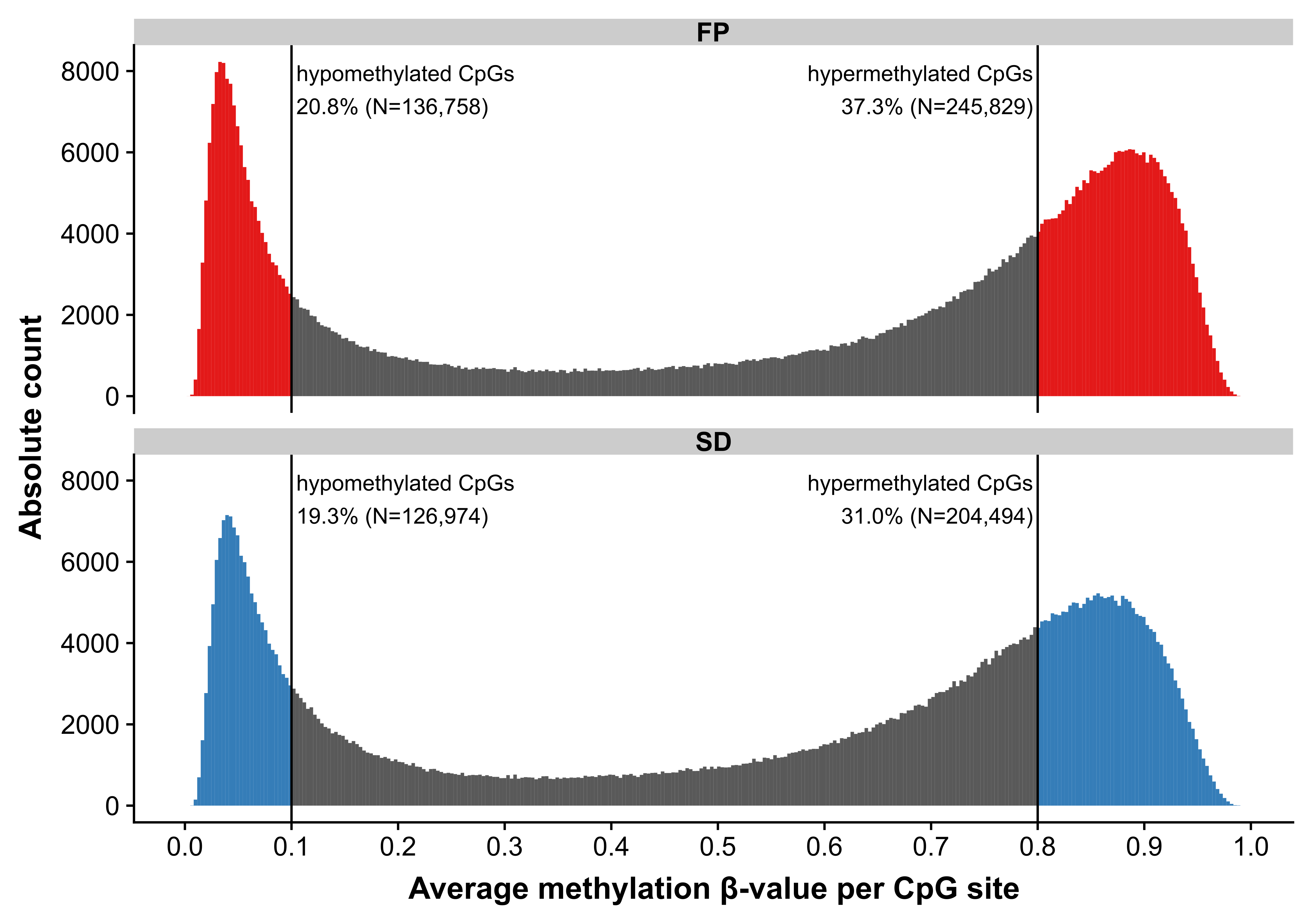

Supplement: Supplementary file 8 — Additional file 8: Figure S6. Histogram of all β-values with fixed thresholds for hyper- and hypomethylation. With a β-value threshold of > 0.8 for hypermethylation and < 0.1 for hypomethylation the simultaneous gain in hyper- and in hypomethylated loci in the fibrotic progression cohort is obvious. The number of CpG sites with very high methylation level (i.e., above 0.8) is larger in the FP cohort compared to the SD cohort (245,829 vs 204,494, or 20.2% more highly methylated sites in the FP cohort) and this increase in highly methylated sites in the FP cohort is three times higher than the increase in CpG sites with very low methylation (i.e., below 0.1) in this cohort (relative to the SD cohort the number of CpG sites with low methylation level increases by only 7.7%). Therefore, it is justified to state that overall the fibrotic progression cohort is characterized by hypermethylation relative to the stable disease cohort. [file 13148_2021_1010_MOESM8_ESM.tif]

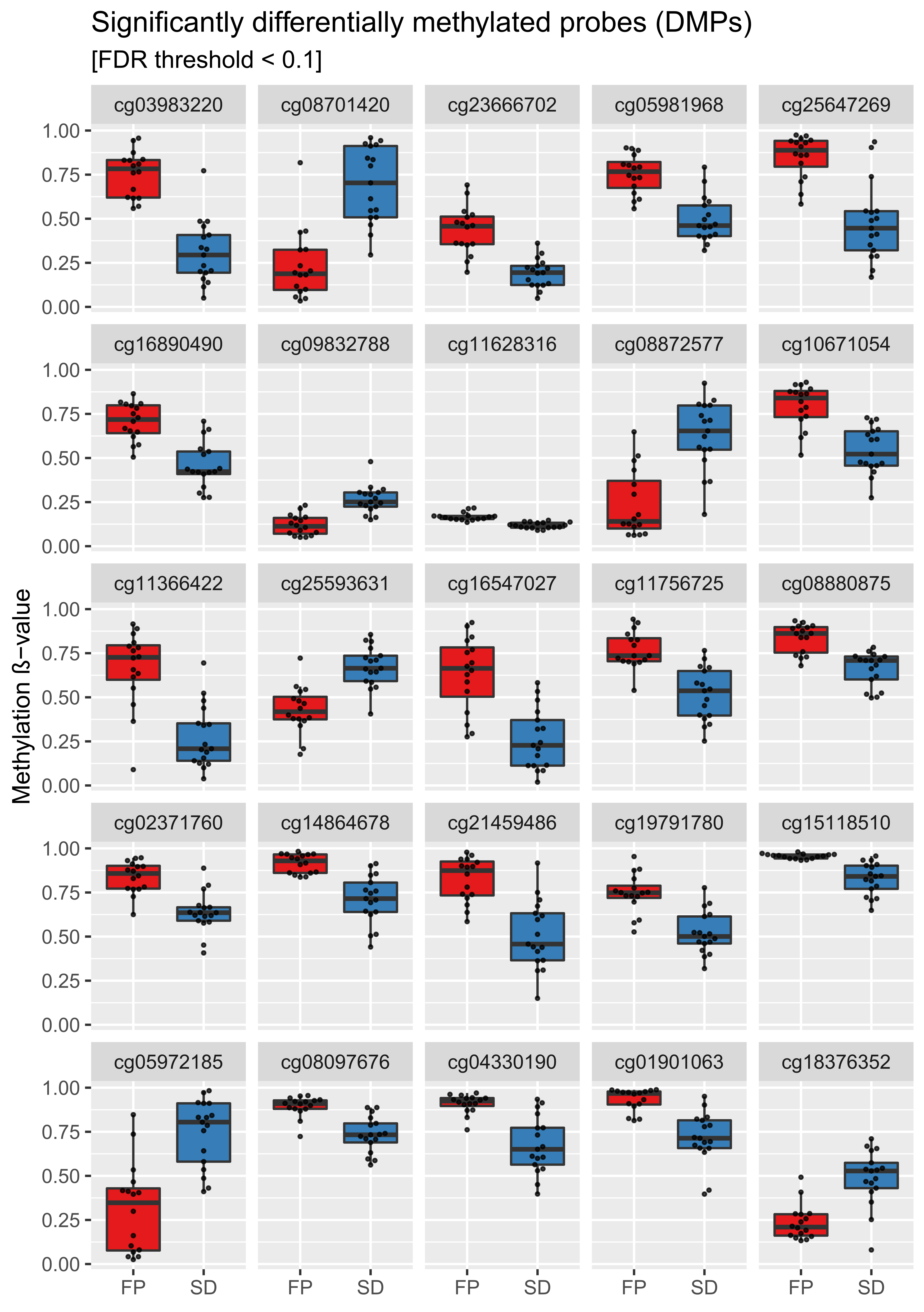

Supplement: Supplementary file 9 — Additional file 9: Figure S7. Boxplots for the β-values of the 25 differentially methylated CpG sites shown in Fig. 1. The display of the individual boxplots shows the simultaneous gain and loss of DNA methylation in the fibrotic progression cohort relative to the stable disease cohort (as shown in a more global view in Additional file 8: Figure S6). 18 from 25 CpG sites display a higher methylation level in the FP group compared to the SD group. [file 13148_2021_1010_MOESM9_ESM.tif]

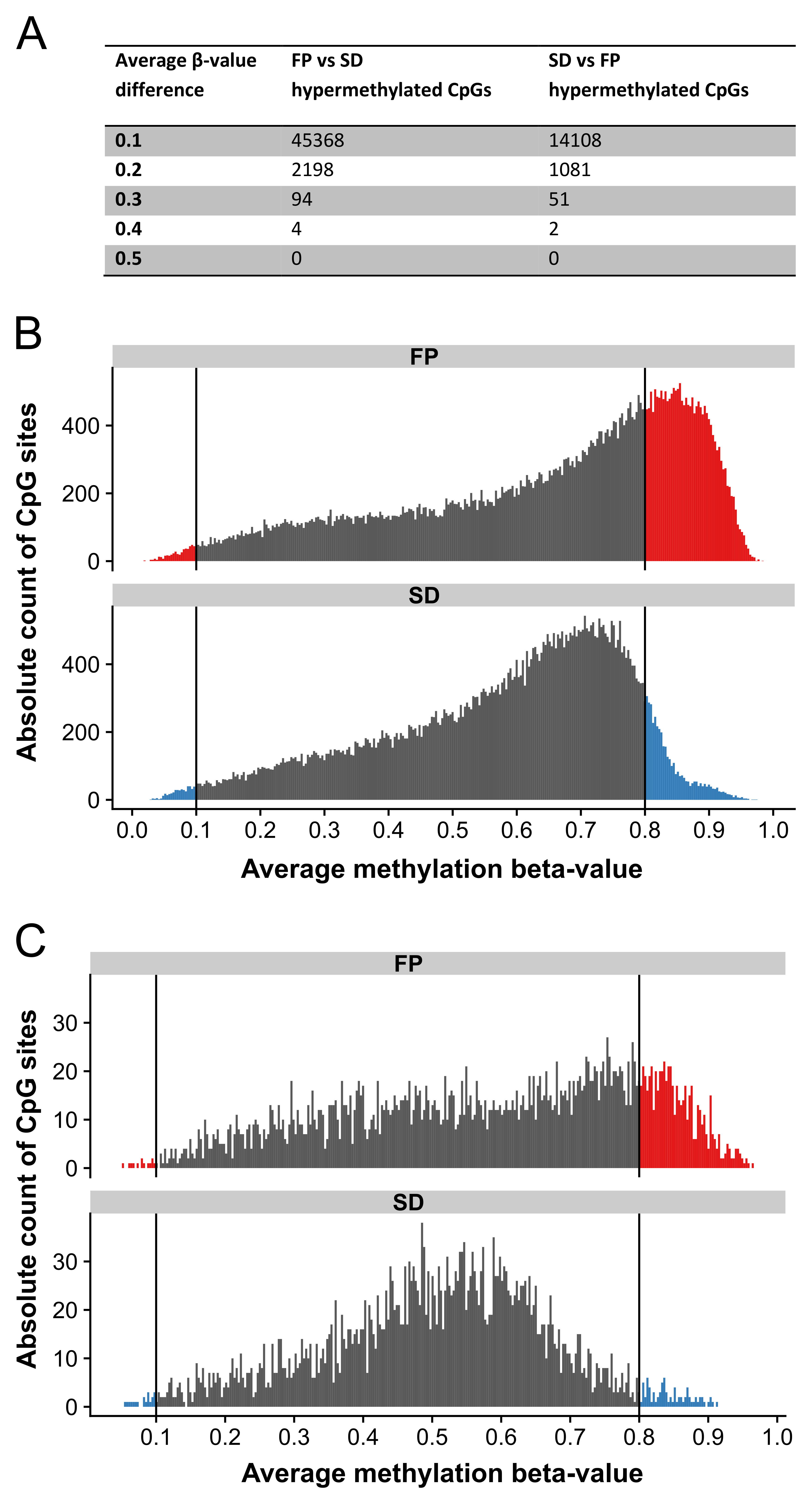

Supplement: Supplementary file 10 — Additional file 10: Figure S8. Number of differentially methylated CpG sites in the FP group versus the SD group. a Number of CpG sites more heavily methylated (“hypermethylated”) in the FP or the SD group at a given threshold for the difference in the β-value. b Histogram for Δβ > 0.1. c Histogram for Δβ > 0.2. The predominance of more heavily methylated loci in the FP group is obvious. The SD group shows a distribution centered around β-values between 0.5 and 0.6, whereas the FP group shows a clear skewing of the distribution towards β-values between 0.8 and 0.9. [file 13148_2021_1010_MOESM10_ESM.tif]

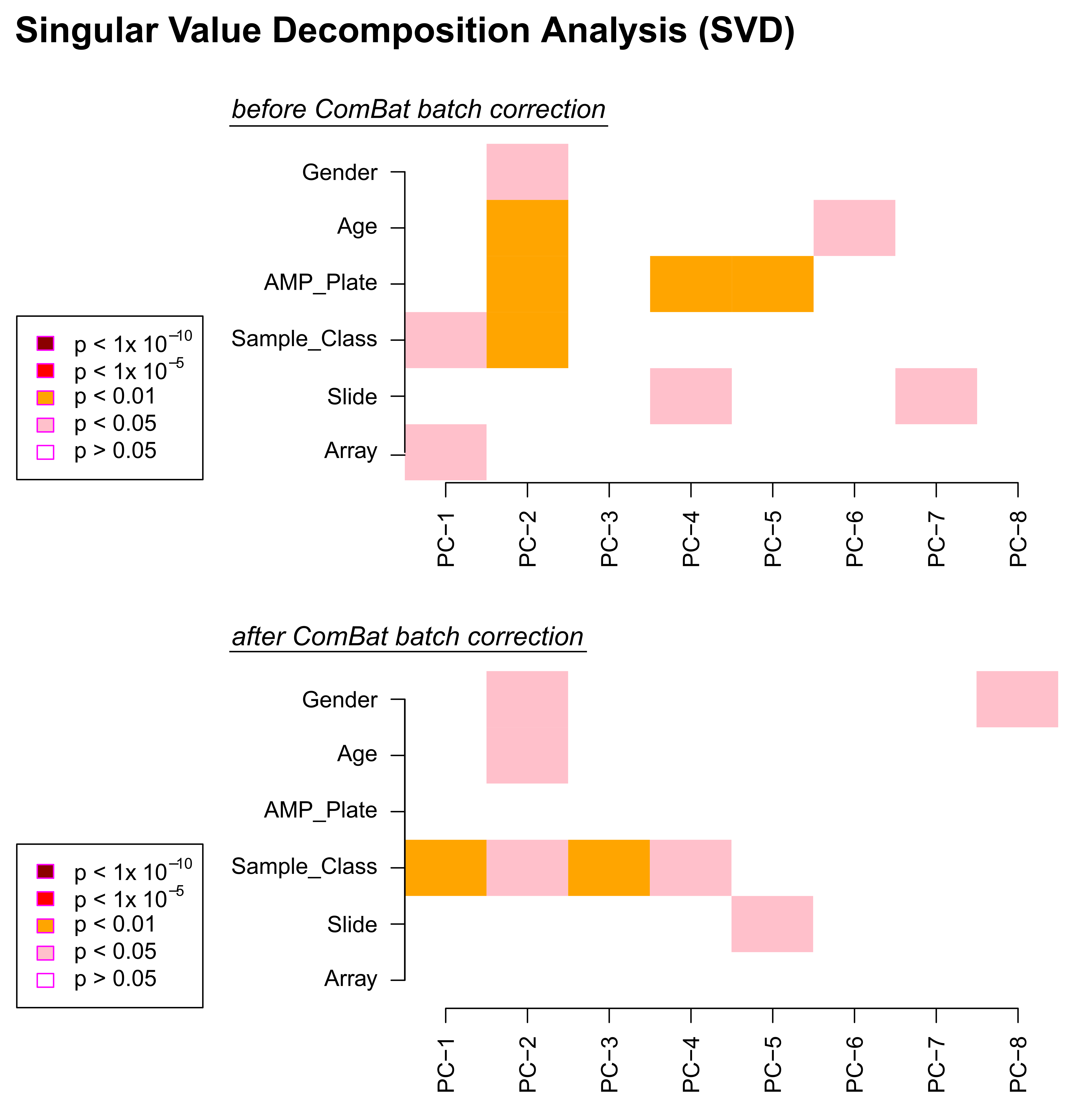

Supplement: Supplementary file 11 — Additional file 11: Figure S9. Singular value decomposition analysis of the batch correction. ComBat batch correction could be successfully used to reduce the influence of confounding variables (such as AMP_Plate) on the methylation data set, leaving “sample class” as the most important discriminator. [file 13148_2021_1010_MOESM11_ESM.tif]
