# Supplementary material for: Genome-wide DNA methylation profiling is able to identify prefibrotic PMF cases at risk for progression to myelofibrosis
Source: Clin Epigenetics. 2021 Feb 4;13:28. doi: 10.1186/s13148-021-01010-y (PMC7860011; doi:10.1186/s13148-021-01010-y)
Supplement: Supplementary file 5 — Additional file 5: Table S1. Gene set enrichment analysis of differentially methylated regions against the Gene Ontology database. [file 13148_2021_1010_MOESM5_ESM.docx]

| **Term** | **Definition** | **Ontology** | **Genes in term** | **Differentially methylated genes** | **P-value** | **FDR** |
| --- | --- | --- | --- | --- | --- | --- |
| O:0007156 | homophilic cell adhesion via plasma membrane adhesion molecules | BP | 152 | 19 | 0 | 0 |
| GO:0098742 | cell-cell adhesion via plasma-membrane adhesion molecules | BP | 231 | 19 | 4.18E-19 | 4.63E-15 |
| GO:0005509 | calcium ion binding | MF | 656 | 20 | 3.46E-13 | 2.56E-09 |
| GO:0098609 | cell-cell adhesion | BP | 776 | 20 | 3.21E-12 | 1.78E-08 |
| GO:0007155 | cell adhesion | BP | 1312 | 24 | 2.17E-11 | 8.86E-08 |
| GO:0022610 | biological adhesion | BP | 1318 | 24 | 2.40E-11 | 8.86E-08 |
| GO:0005887 | integral component of plasma membrane | CC | 1524 | 23 | 7.30E-11 | 2.31E-07 |
| GO:0031226 | intrinsic component of plasma membrane | CC | 1582 | 23 | 1.64E-10 | 4.55E-07 |
| GO:0007267 | cell-cell signaling | BP | 1515 | 23 | 1.30E-09 | 3.19E-06 |
| GO:0046872 | metal ion binding | MF | 3897 | 33 | 6.66E-09 | 1.47E-05 |
| GO:0043169 | cation binding | MF | 3979 | 33 | 9.88E-09 | 1.99E-05 |
| GO:0043167 | ion binding | MF | 5795 | 38 | 1.95E-07 | 3.60E-04 |
| GO:0007399 | nervous system development | BP | 2140 | 25 | 2.47E-07 | 4.21E-04 |
| GO:0044459 | plasma membrane part | CC | 2495 | 24 | 9.49E-07 | 1.50E-03 |
| GO:0031224 | intrinsic component of membrane | CC | 5065 | 29 | 2.38E-05 | 3.51E-02 |
| GO:0007275 | multicellular organism development | BP | 4932 | 32 | 2.63E-05 | 3.64E-02 |
| GO:0048731 | system development | BP | 4403 | 30 | 3.27E-05 | 4.25E-02 |
| GO:0016021 | integral component of membrane | CC | 4938 | 28 | 4.26E-05 | 5.24E-02 |
| GO:0048856 | anatomical structure development | BP | 5387 | 33 | 4.95E-05 | 5.76E-02 |
| GO:0032502 | developmental process | BP | 5769 | 34 | 6.34E-05 | 7.01E-02 |
